# Neurobehavioral dysregulation, depressive symptoms, and cognition in former American football players

**DOI:** 10.3389/fneur.2026.1952836

**Published:** 2026-10-13

**Authors:** Brooke D. Conway Kleven, Michael L. Alosco, Jennifer V. Wethe, Sarah J. Banks, Charles H. Adler, Douglas I. Katz, Robert C. Cantu, Laura J. Balcer, Yorghos Tripodis, Jesse Mez, Charles Bernick, Eric M. Reiman, Robert A. Stern, Jeffrey L. Cummings

**Affiliations:** 1 Department of Epidemiology and Biostatistics, School of Public Health, University of Nevada, Las Vegas, Las Vegas, NV, United States; 2 Department of Brain Health, Kirk Kerkorian School of Medicine, University of Nevada, Las Vegas, Las Vegas, NV, United States; 3 Chambers-Grundy Center for Transformative Neuroscience, Department of Brain Health, Kirk Kerkorian School of Medicine, University of Nevada, Las Vegas, Las Vegas, NV, United States; 4 Department of Neurology, Boston University Chobanian & Avedisian School of Medicine, Boston, MA, United States; 5 Boston University Alzheimer’s Disease Research Center, Boston, MA, United States; 6 BU CTE Center, Boston University, Boston, MA, United States; 7 Department of Anatomy & Neurobiology, Boston University Chobanian & Avedisian School of Medicine, Boston, MA, United States; 8 Department of Neurology, Boston Medical Center, Boston, MA, United States; 9 Department of Psychiatry and Psychology, Mayo Clinic School of Medicine and Science, Scottsdale, AZ, United States; 10 Department of Neurosciences, University of California San Diego School of Medicine, San Diego, CA, United States; 11 Department of Psychiatry, University of California San Diego School of Medicine, San Diego, CA, United States; 12 Department of Neurology, Mayo Clinic School of Medicine and Science, Scottsdale, AZ, United States; 13 Encompass Health Rehabilitation Hospital, Braintree, MA, United States; 14 Department of Neurology, Grossman School of Medicine, New York University, New York, NY, United States; 15 Center for Health Data Science, Boston University School of Public Health, Boston, MA, United States; 16 Cleveland Clinic Lou Ruvo Center for Brain Health, Las Vegas, NV, United States; 17 Banner Alzheimer’s Institute, Phoenix, AZ, United States; 18 Department of Psychiatry, University of Arizona College of Medicine—Phoenix, Phoenix, AZ, United States

**Keywords:** American football, cognition, depression, neurobehavioral dysregulation, neurotrauma, repetitive head impacts, sports, traumatic encephalopathy syndrome

## Abstract

Neurobehavioral dysregulation (NBD) is a core clinical feature of traumatic encephalopathy syndrome (TES), but its relationships with repetitive head impact (RHI) exposure, cognitive performance, and depressive symptoms remain unclear. This study examined clinical factors associated with NBD among former American football players with substantial RHI exposure. Cross-sectional baseline data were analyzed from 179 former American football players enrolled in the DIAGNOSE CTE Research Project, including 119 former professional and 60 former college players. NBD was assessed using measures of impulsivity, behavioral regulation, hostility, and emotional lability. Multivariate regression models evaluated associations of continuous NBD scores with depressive symptom dimensions, cognitive performance, playing level, and years of football participation, adjusting for demographic and clinical covariates. Secondary analyses evaluated dichotomized NBD outcomes using study-specific thresholds. Bonferroni-adjusted *p*-values were reported for multiple comparisons. Depressive symptoms demonstrated the most consistent associations with NBD. Greater neurovegetative depressive symptom burden was associated with greater impulsivity (estimate = 6.84; 95% CI = 4.45, 9.23; *p*-value < 0.0001), behavioral dysregulation (estimate = 11.63; 95% CI = 8.50, 14.76; *p*-value < 0.0001), hostility (estimate = 5.07; 95% CI = 2.32, 7.81; *p*-value = 0.0004), and emotional lability (estimate = 1.16; 95% CI = 0.12, 2.20; *p*-value = 0.0284). Greater agitated/reactive depressive symptom burden was associated with greater impulsivity (estimate = 3.42; 95% CI = 1.44, 5.41; *p*-value = 0.0008) and hostility (estimate = 5.11; 95% CI = 2.89, 7.32; *p*-value < 0.0001), while greater affective depressive symptom burden was associated with greater behavioral dysregulation (estimate = 3.09; 95% CI = 0.01, 6.18; *p*-value = 0.0494). Better verbal memory was associated with lower impulsivity (estimate = −2.02; 95% CI = -3.65, −0.40; *p*-value = 0.0149) and behavioral dysregulation (estimate = −2.64; 95% CI = −4.76, −0.52; *p*-value = 0.0150), while better executive speed was associated with lower behavioral dysregulation (estimate = −3.10; 95% CI = −5.11, −1.09; *p*-value = 0.0027). Years of football exposure was not independently associated with NBD outcomes. Among former American football players with substantial RHI exposure, depressive symptom burden was more consistently associated with NBD than football participation characteristics or cognitive performance. These findings support a heterogeneous clinical phenotype and underscore the importance of evaluating depressive symptoms when interpreting NBD in the context of TES.

## Introduction

Frequent exposure to head impacts in contact sport athletes is a growing concern due to the associated risk for neurocognitive and neurobehavioral disorders (1–3). In some cases, repetitive head impact (RHI) exposure has been shown to lead to chronic traumatic encephalopathy (CTE) (4). The clinical syndrome posited to be associated with the pathological diagnosis of CTE, traumatic encephalopathy syndrome (TES), is characterized by (1) substantial exposure to RHI, (2) a core clinical feature of cognitive impairment and/or neurobehavioral dysregulation (NBD), (3) symptoms showing a progressive course, and (4) symptoms not explained by other disorders (5). NBD, although one of the core clinical features of TES, is broadly defined. The National Institute of Neurological Disorders and Stroke (NINDS) clinical criteria define NBD using six neuropsychiatric descriptors, but there remains overlap with other disorders such as behavioral variant of frontotemporal dementia (FTD), intermittent explosive disorder, and frontal variant of Alzheimer’s disease (AD) (6–8).

There is a lack of validated diagnostic tools to characterize NBD. Scales and subscales from multiple neuropsychiatric instruments have been used in studies of the phenotype of NBD. Analysis of data from the Diagnostics, Imaging, and Genetics Network for the Objective Study and Evaluation of Chronic Traumatic Encephalopathy (DIAGNOSE CTE) of former American football players revealed four neuropsychiatric subconstructs that characterized NBD (9). This symptom profile included explosivity (sudden outbursts of anger or aggression disproportionate to the provocation), emotional dyscontrol (inability to regulate emotions), impulsivity (tendency to act without considering consequences), and affective lability (variability in the outward display of emotion, such as crying disproportionate to sadness) (9). As a diagnosis-specific instrument which encompasses these four subconstructs is not yet available, validating subscales of various neuropsychiatric assessments may help capture the symptoms characteristic of NBD. Improved characterization can facilitate detection and differential diagnosis of NBD and TES, and may help distinguish the NBD syndrome from behavioral changes associated with other neurodegenerative and psychiatric disorders.

Recent findings suggest that NBD may emerge earlier in the clinical course than cognitive impairment among athletes with RHI exposure, with younger individuals demonstrating greater impulsivity and behavioral dysregulation (9). Furthermore, these symptoms are not specific to underlying CTE pathology and may arise from multiple contributing mechanisms (10). Specifically, neuropsychiatric symptoms such as impulsivity, emotional dyscontrol, and affective lability may reflect a heterogeneous clinical construct rather than a direct or singular manifestation of CTE. These symptoms may be influenced by multiple factors, including neuroinflammation, neuronal dysfunction/neurodegeneration, pre-existing individual characteristics, personality traits, sensation seeking, risk-taking propensity, and other non-CTE processes (11, 12). Consequently, these symptoms may arise through multiple pathways and may not be specific indicators of underlying CTE pathology. This clinical heterogeneity raises important questions regarding whether NBD represents a core feature of TES or is a nonspecific syndrome that may precede and/or co-occur with cognitive impairment.

Depressive symptom burden may represent one factor contributing to the observed heterogeneity in NBD. Depression is closely linked to dysregulation of stress response systems, including hypothalamic–pituitary–adrenal axis activation, chronic inflammation, and autonomic imbalance, which collectively contribute to increased allostatic load (the cumulative physiological burden on the body resulting from chronic stress and repeated activation of stress response systems) (13). In the NINDS criteria for TES, depression is noted as a supportive feature for TES but is not included as a core feature of the syndrome (5). However, depression remains clinically relevant in RHI-exposed populations, particularly given emerging evidence that former professional football players with perceived CTE exhibit substantially greater depressive symptom burden and suicidality (14). In this context, depression may represent an important contributor to neuropsychiatric symptom burden while also carrying implications for clinical risk assessment and safety monitoring.

The purpose of this study was to evaluate the relationship between NBD and neurocognitive performance in former American football players with substantial RHI exposure, and to determine how depressive symptom burden influences these relationships. Given the overlap between neuropsychiatric symptoms, cognitive function, and mood disturbance in RHI-exposed populations, we examined whether associations between NBD and neurocognitive performance varied by exposure characteristics, including playing level and football participation history, while accounting for depressive symptoms and other clinical factors. We examined the relationship between NBD and the diagnosis of TES. This integrative framework extends prior work by jointly considering neuropsychiatric function, cognition, mood, and exposure context, rather than evaluating these features in isolation. We hypothesized that poorer neurocognitive performance and greater RHI exposure would be associated with higher NBD severity, and that depressive symptom burden would independently contribute to NBD and potentially influence observed relationships between cognition and NBD.

## Materials and methods

### Study participants

Methods for the DIAGNOSE CTE Research Project have been described in detail (15). In brief, this eight-year longitudinal study recruited 240 men ages 45 to 74, including 120 former professional football players (PRO), 60 former college football players (COL), and 60 asymptomatic males with no history of participation in organized contact/collision sports or other activities associated with RHI [unexposed (UE)]. For purposes related to this study, we have excluded the UE controls without RHI exposure and examine baseline data only. For the PRO and COL groups, there were no specific inclusion or exclusion criteria regarding presence or severity of cognitive impairment, neuropsychiatric symptoms, functional independence, or motor disorder, although participants were required to have adequate decisional capacity to provide consent. All participants had a study partner who completed research assessments. To be included in the cohort, the PRO participants were required to have played 12 or more years of organized football, including three or more years in college and more than 3 years in the National Football League (NFL). COL participants were required to have played six or more years of American football with three or more years at the college level. A national recruitment campaign aimed at enrolling PRO and COL participants across a continuum of symptom severity from asymptomatic to mildly symptomatic to dementia, without any prespecified level or type of impairment. Interested study candidates underwent a telephone interview with structured and semi-structured questions regarding mood, behavior, cognition, and functional status. A separate telephone screening was conducted with the study partner. Baseline evaluations were comprehensive and included neurocognitive testing, assessment of functional status, administration of neuropsychiatric questionnaires, neurological examination, structural and molecular imaging, and fluid-based biomarkers. A consensus conference adjudicated all participants for a research diagnosis of TES per the National Institute of Neurological Disorders and Stroke clinical criteria (15). TES adjudication processes, cognitive assessments, and neuropsychiatric results have been reported in detail (5, 15–17).

### Characterization of NBD, cognitive function, depression, and RHI exposure

The full battery of neuropsychiatric assessments used to evaluate the core features of NBD among participants in DIAGNOSE CTE included the Barratt Impulsiveness Scale-11 (BIS-11) (10, 18); Behavior Rating Inventory of Executive Function—Adult Version (BRIEF-A) Behavioral Regulation Index (BRI) (19); Brown-Goodwin Lifetime History of Aggression (BGLHA Childhood, Adolescence, Adulthood) (19, 20); Buss-Durkee Hostility Inventory (BDHI) (21, 22); Center for Neurologic Study Lability Scale (CNS-LS) (21, 23); Neuropsychiatric Inventory-Questionnaire (NPI-Q) (23, 24); and the State–Trait Anger Expression Inventory-2 (STAXI-2) (24–26). The content of these scales, supportive scales, and the scoring parameters for all instruments included in the DIAGNOSE CTE battery are provided in Table 1.

**Table 1:** Neuropsychiatric and behavioral scales administered in DIAGNOSE CTE research project.

Instrument	Brief description	Scoring^*^
Scales for core behaviors		
Barratt Impulsiveness Scale-11 (BIS-11)	Self-report questionnaire assessing attentional, motor, and non-planning impulsivity	Total scores are typically interpreted dimensionally, with higher scores indicating greater impulsivity. In adult normative data, scores ≥72 have been used to indicate high impulsivity, while scores 52–71 fall within the typical adult range.
Behavior Rating Inventory of Executive Function (BRIEF) Total	Self- and/or informant-report measure of real-world executive functioning	Scores are reported as age-adjusted T-scores; higher scores indicate greater executive dysfunction. T-scores ≥65 are considered clinically elevated.
BRIEF Behavioral Regulation Index (BRI)	Index reflecting inhibition, shifting, emotional control, and self-monitoring	Scores are reported as age-adjusted T-scores; higher scores indicate greater behavioral regulation difficulty. T-scores ≥65 are considered clinically elevated.
Brown-Goodwin Lifetime History of Aggression (BGLHA Childhood)	Assesses aggressive acts occurring in childhood	Scores are interpreted dimensionally; higher scores indicate greater lifetime aggression during childhood.
Brown-Goodwin Lifetime History of Aggression (BGLHA Adolescence)	Assesses aggressive acts occurring in adolescence	Scores are interpreted dimensionally; higher scores indicate greater lifetime aggression during adolescence.
Brown-Goodwin Lifetime History of Aggression (BGLHA Adulthood)	Assesses aggressive acts occurring in adulthood	Scores are interpreted dimensionally; higher scores indicate greater lifetime aggression during adulthood.
Buss-Durkee Hostility Inventory (BDHI)	75-item true/false measure assessing hostility and aggression-related tendencies	Total scores range from 0–75, with higher scores indicating greater hostility. No widely accepted clinical cutoff has been established; scores are typically interpreted dimensionally or using study-specific thresholds.
STAXI-2 State Anger	Assesses intensity of anger as a current emotional state	Scores are interpreted using normative T-scores and percentiles; higher scores indicate greater state anger.
STAXI-2 Trait Anger	Assesses disposition to experience anger over time	Scores are interpreted using normative T-scores and percentiles; higher scores indicate greater trait anger.
STAXI-2 Anger Expression Index	Assesses anger expression and control patterns	Scores are interpreted using normative T-scores and percentiles; higher scores indicate greater anger expression dysregulation.
Scales for supportive behaviors		
Apathy Evaluation Scale (AES)	18-item self-report assessing cognitive, emotional, and behavioral aspects of apathy	Scores range from 18–72, with higher scores indicating greater apathy. Published cutoffs vary by population; no single universal cutoff applies. Prior work in this population cites a cutoff score of 34.
Beck Anxiety Inventory (BAI)	21-item self-report measure of anxiety symptom severity	Total scores range from 0–63: 0–7 minimal, 8–15 mild, 16–25 moderate, and 26–63 severe anxiety.
Beck Depression Inventory-II (BDI-II)	21-item self-report measure of depressive symptom severity	Total scores range from 0–63: 0–13 minimal, 14–19 mild, 20–28 moderate, and 29–63 severe depression.
Beck Hopelessness Scale (BHS)	20-item self-report measure of hopelessness and negative expectations about the future	Total scores range from 0–20; higher scores indicate greater hopelessness. Severity bands commonly include 0–3 minimal, 4–8 mild, 9–14 moderate, and 15–20 severe hopelessness. Scores ≥8 or ≥9 have been used to indicate elevated suicide risk, depending on study/population.
Center for Neurologic Study-Lability Scale (CNS-LS)	7-item self-report measure of affective lability	Total scores range from 7–35, with higher scores indicating greater affective lability. A cutoff of ≥13 has been used to suggest pseudobulbar affect in ALS, and higher cutoffs such as ≥17 have been used in MS/ALS contexts; these thresholds are disease-specific and not validated for RHI-exposed athletes.
Neuropsychiatric Inventory-Questionnaire (NPI-Q)	Informant-based measure of neuropsychiatric symptoms in neurologic disorders	Severity scores range from 0–36, with higher scores indicating greater neuropsychiatric symptom severity. No universal clinical cutoff applies across populations.
Sheehan-Suicidality Tracking Scale (Sheehan-SST)	Assesses severity and change in suicidal ideation and behavior	Scores are used to characterize and track suicidality severity over time. It is not typically interpreted using a single diagnostic cutoff; change thresholds may be used for longitudinal monitoring.
UCLA Loneliness Scale	Self-report measure of perceived loneliness and social isolation	Scores are typically interpreted dimensionally, with higher scores indicating greater loneliness. Published thresholds vary across studies.
Montreal Cognitive Assessment (MoCA)	30-point cognitive screening measure	Total scores range from 0–30. The original validation used <26 as abnormal, although lower cutoffs may improve specificity in some populations.
Unified Parkinson’s Disease Rating Scale (UPDRS), Part III	Clinician-rated assessment of motor signs of parkinsonism	Scores are interpreted dimensionally, with higher scores indicating greater motor impairment. No universal diagnostic cutoff applies; severity interpretation depends on clinical context and Parkinson disease stage.

^*^Published scoring thresholds or interpretive guidance are provided where available. Several scales do not have validated clinical cutoffs and are interpreted dimensionally.

The Beck Depression Inventory-II (BDI) is a 21-item self-report questionnaire used to assess the severity of depressive symptoms over the preceding 2 weeks (26, 27). Each item is rated on a 4-point scale (0–3), with total scores ranging from 0 to 63, where higher scores indicate greater depressive symptom severity. The BDI captures cognitive and somatic (neurovegetative) symptoms of depression and has been widely validated across clinical and research populations (28).

Cognitive factor scores were derived using standardized neuropsychological test performance and represent latent domains of cognitive functioning previously characterized in DIAGNOSE CTE analyses (16, 28). Verbal memory reflects learning and delayed recall of verbally presented information, capturing episodic memory processes. Visual memory reflects the encoding and recall of visuospatial information. Number span represents attention and working memory capacity, particularly the ability to temporarily store and manipulate numeric information. Verbal fluency reflects language production and executive retrieval processes, including the ability to generate words under phonemic or semantic constraints. Higher factor scores indicate better cognitive performance within each domain.

Participant exposure history, including age of first exposure, level(s) and duration of play, and position(s) played, was used to derive estimates of cumulative head impact exposure (CHII). CHII integrates self-reported football history with validated head impact exposure metrics derived from instrumented helmet data to generate participant-specific estimates of cumulative exposure burden, consistent with previously published methods from DIAGNOSE CTE (15, 27).

### Primary outcome measures

Based on prior analyses within this cohort, the BIS-11 (impulsivity), BRI (behavioral regulation), BDHI (hostility), and CNS-LS (emotional lability) were identified as the four primary outcome measures for this analysis (9, 28). Instrument scores were examined in two complementary formats as described in detail in the *Statistical Analyses*. Continuous scores were analyzed as the primary approach to capture the full distribution of responses and to evaluate associations with neurocognitive performance, depressive symptoms, RHI exposure, and TES. Secondary exploratory analyses examined dichotomized NBD outcomes to characterize participants with relatively greater neurobehavioral symptom burden. Because validated clinical thresholds are unavailable for most NBD instruments in former football players, empirically derived study-specific cut points were evaluated and compared with published thresholds where available.

### Statistical analyses

Initial evaluations included descriptive statistics of the population and comparisons between exposure groups (PRO and COL) using analysis of variance for continuous variables (cognitive factor scores, education, NBD scale scores, BDI total score, years of football, lifetime head acceleration exposure, and years since retirement) and Fisher–Freeman–Halton test for categorical variables (position played, TES status, pre-existing history of attention disorders, age group, and race). Age was categorized into decade-based groups to protect participant confidentiality, given that many participants are publicly identifiable former athletes. Pearson’s correlations were performed to evaluate the relationship between RHI exposure and each of the four NBD instrument scale scores. Cumulative linear and rotational head acceleration were also evaluated. As these metrics did not provide additional information or materially alter model interpretation, total years of football exposure was retained as the primary exposure measure in adjusted models for simplicity and interpretability.

The NBD outcomes were examined primarily as continuous variables. Secondary analyses evaluated dichotomized NBD outcomes using empirically derived cut points because validated clinical thresholds have not been established for most instruments in former football players or RHI-exposed populations. For binary analyses, optimal thresholds were empirically derived for each NBD scale using statistical criteria to identify cut points that maximized discrimination between participants classified as TES+ with NBD versus all other participants, including TES− participants and TES+ participants with cognitive impairment only (15, 29). Specifically, all candidate cut points (*k* − 1, where *k* denotes the number of unique values) were evaluated, and the cut point yielding the lowest *p*-value and largest test statistic was selected. Following threshold identification, NBD scale scores were dichotomized as above versus below each cut point. These thresholds were used to characterize symptom burden within this cohort and were not intended to establish diagnostic or clinically validated cut points. Table A1 in the Appendix provides a comparison of empirical cut points to published clinical thresholds where available.

Binary outcomes were modeled using multivariate Firth penalized logistic regression. Models evaluated whether global depressive symptom severity (BDI total score) accounted for the relationship between the five cognitive factor scores and NBD outcomes, adjusting for age, race, pre-existing attention disorders, exposure classification (PRO/COL), years of education, and total years of football participation. Model discrimination was evaluated through the use of receiver operating characteristic (ROC) curves, and model fit was assessed using lack-of-fit tests and pseudo-R^2^ statistics. Multicollinearity was evaluated using variance inflation factors, tolerance statistics, and condition indices. No evidence of problematic multicollinearity was observed.

To examine whether distinct characteristics of depressive symptoms were associated with NBD outcomes, exploratory factor analysis was applied to individual BDI items using maximum likelihood estimation with oblique (promax) rotation to allow for correlated symptom domains. Factor retention was guided by eigenvalues, scree plot inspection, and factor interpretability. Three distinct depressive factor scores were retained based on overall model coherence and were incorporated into subsequent analyses. NBD outcomes were then examined as continuous variables using multivariate regression models. Separate models were fit for each of the four NBD outcomes (BIS-11, BRI, BDHI, and CNS-LS), with depressive and cognitive factor scores evaluated simultaneously. Models adjusted for age, race, pre-existing attention disorders, exposure classification, years of education, and total years of football participation. A sensitivity analysis was evaluated for effect modification by depressive symptom burden by including interaction terms between centered years of football participation and centered BDI total score in multivariate regression models for each neurobehavioral outcome. All analyses were conducted using SAS software (version 9.4; SAS Institute Inc., Cary, NC). Bonferroni correction was applied for multiple comparisons, with statistical significance defined as *p*-value < 0.05 after correction.

## Results

### Demographic characteristics

The study sample included 179 participants (119 PRO, 60 COL), as 1 participant (1 PRO) was excluded from the current analyses due to missing necessary data. Of the full sample, 65% were TES+, including 53% who were TES+ with NBD (with or without cognitive impairment) and 12% who were TES+ with cognitive impairment only. No significant differences in TES diagnosis were observed between groups (*p*-value = 0.0679). Depressive symptom severity also did not differ significantly between PRO and COL groups (*p*-value = 0.0898). Educational attainment was modestly lower among PRO players (*p*-value = 0.0368). None of the core NBD scale scores, including impulsivity, behavioral regulation, hostility/aggression, and emotional lability, differed significantly between PRO versus COL (all *p*-values >0.05). COL participants had a greater percentage of participants with pre-existing attention disorders (COL = 15%; PRO = 5%; *p*-value = 0.0415) and a lower percentage of black/African American participants than PRO participants (COL = 18.6%; PRO = 43.6%; *p*-value = 0.0020). PRO participants were relatively older at the time of evaluation (*p*-value < 0.0001).

Exposure-related characteristics differed substantially between groups. As expected from the group definitions, PRO reported significantly more years of football participation (*p*-value < 0.0001) and greater estimated lifetime head impact exposure load (*p*-value < 0.0001). COL reported a greater number of seasons of tackle football prior to high school (*p*-value = 0.0074) and a longer interval between football retirement and baseline evaluation (*p*-value = 0.0480). No significant differences were observed between groups in relation to playing position (*p*-value = 0.2284). More complete details of study demographics are provided in Tables 2, 3.

**Table 2:** Descriptive statistics of demographic variables among professional (PRO) and college (COL) American football players.

Characteristic	Group				
	PRO(*N* = 119)		COL(*N* = 60)		
	*N*	%	*N*	%	*p*-value
Position group at highest level of football					0.2284
Linemen	36	20.1	27	15.1	
Offensive backs and receivers	37	20.7	14	7.8	
Linebackers	22	12.3	7	3.9	
Defensive backs	24	13.4	12	6.7	
TES status					0.0679
TES+	83	46.4	33	18.4	
TES+ with NBD^*^	64	35.8	31	17.3	
TES−	36	20.1	27	15.1	
Pre-existing history of attention disorders					0.0415
Yes	6	3.4	9	5.0	
No	113	63.1	51	28.5	
Age group					<0.0001
45–54	41	17.5	44	18.7	
55–64	46	19.6	9	3.8	
65–74	32	13.6	7	3.0	
Race					0.002
Black/African American	51	28.5	11	6.2	
White	66	36.9	48	26.8	

^*^TES+ with NBD is not a separate group but a subset of TES+ participants. Of the 116 TES+ participants, 95 participants had TES+ with NBD (with or without cognitive impairment) and 21 participants had cognitive impairment only without NBD.

**Table 3:** Descriptive statistics of cognitive, NBD, and exposure scores among professional (PRO) and college (COL) American football players.

Characteristic	Group				
	PRO(*N* = 119)		COL(*N* = 60)		
	Mean	SD	Mean	SD	*p*-value
Cognitive measures					
MoCA (raw score)	24.3	3.5	25.4	3.2	0.0563
WRAT-4 (standardized score)	100.2	13.4	106.0	12.0	0.0054
Verbal memory (factor score)	−0.3	0.9	0.1	1.0	0.0117
Executive speed (factor score)	0.0	1.1	−0.1	0.9	0.6108
Visual memory (factor score)	−0.1	1.0	0.3	0.9	0.0360
Number span (factor score)	0.0	0.9	0.0	1.0	0.9155
Verbal fluency (factor score)	0.0	0.9	0.1	1.1	0.5099
BDI (scale score)	11.5	9.4	14.2	11.0	0.0898
Educational attainment (years)	16.6	1.1	17.1	1.9	0.0368
Core behaviors of NBD					
BIS-11 (scale score)	64.3	13.9	67.1	14.1	0.2124
BRIEF BRI (scale score)	67.7	19.6	70.5	19.8	0.3605
BDHI (scale score)	27.6	13.7	31.8	14.7	0.0621
CNS-LS (scale score)	12.8	4.4	12.2	4.6	0.3854
Exposure scores					
Total football play (years)	18.0	3.3	11.5	2.5	<0.0001
Lifetime Load: Football Hits based on PEM linear acceleration (g-force)	188,610.2	49,588.5	138,508.4	42,835.5	<0.0001
Number of seasons of tackle football prior to high school	3.5	2.0	4.4	2.0	0.0074
Years between football retirement and baseline evaluation	28.5	8.6	31.1	8.1	0.0480

Stratifying PRO/COL exposure groups by TES status showed that COL players had significantly higher mean scores on the BDI and three of the four NBD measures (BIS-11, BRI, and BDHI) compared with PRO participants. TES+ participants also had significantly higher scores than TES− participants across all five measures (all *p*-values ≤ 0.0011). The exposure × TES interaction was not significant for any measure (all *p*-values ≥ 0.49), indicating that PRO and COL participants differed overall, but TES affected both groups in a similar way. Conversely, CNS-LS scores did not differ between COL and PRO (*p*-value = 0.8348), although scores were higher among TES+ than TES− participants (*p*-value = 0.0011) (Table 4). Among TES+ participants, COL players had significantly higher BDI scores than PRO players (18.58 vs. 14.72; *p*-value = 0.0331). A similar difference was observed among TES− participants (COL = 8.85 vs. PRO = 4.11; *p*-value = 0.0340). Cognitive impairment was also less common in the COL than PRO participants who met TES criteria (57.6% vs. 83.1%; *p*-value = 0.0037).

**Table 4:** NBD scale scores stratified by exposure groups and TES adjudication.

Scale	Group										
	PRO				COL						
	TES+		TES−		TES+		TES−				
	Mean	SD	Mean	SD	Mean	SD	Mean	SD	*p*_1_	*p*_2_	*p*_3_
BIS-11	68.78	12.79	53.89	10.44	72.88	12.58	60.04	12.74	0.0123	<0.0001	0.6128
BRI	75.01	17.52	50.67	12.19	80.79	16.57	57.93	15.92	0.0147	<0.0001	0.7796
BDHI	31.16	13.91	19.63	9.23	38.33	13.95	23.89	11.53	0.0072	<0.0001	0.4900
CNS-LS	13.65	4.35	10.86	3.75	13.06	5.14	11.15	3.58	0.8348	0.0011	0.5353
BDI	14.72	9.29	4.11	3.87	18.58	10.52	8.85	9.08	0.0030	<0.0001	0.7560

^*^p-values are reported as: *p*_1_ = exposure; *p*_2_ = TES; *p*_3_ = exposure × TES interaction.

### Multivariate predictors of NBD

*Depressive severity*. Adjusting for age, exposure burden, pre-existing attention disorders, education, and race in Firth penalized logistic regression models, greater depressive symptom severity, as measured by the BDI total score, was consistently associated with increased odds of higher NBD symptom burden across all four NBD behavioral domains (Table 5). For impulsivity (BIS-11), each one-point increase in BDI score was associated with a 13% increase in the odds of elevated impulsivity (OR = 1.13; 95% CI = 1.08, 1.19; *p*-value < 0.0001). Similar associations were observed for behavioral regulation at 19% (BRI OR = 1.19; 95% CI = 1.12, 1.26; *p*-value < 0.0001), hostility/aggression at 10% (BDHI OR = 1.10; 95% CI = 1.06, 1.16; *p*-value < 0.0001), and emotional lability at 8% (CNS-LS OR = 1.08; 95% CI = 1.03, 1.12; *p*-value = 0.0007).

**Table 5:** Adjusted logistic regressions modeling the odds of higher NBD scale scores.

Effect	OR	95% CI		*p*-value
BIS-11				
Age (45–54 vs. 65–74)	4.14	1.06	16.17	0.0410
Age (55–64 vs. 65–74)	1.00	0.24	4.11	0.9972
Exposure (COL vs. PRO)	1.03	0.25	4.23	0.9702
Pre-existing attention disorder (Yes vs. No)	0.75	0.17	3.30	0.6982
Educational attainment	1.03	0.72	1.47	0.8669
Years of football play	1.01	0.87	1.17	0.9323
BDI Scale Score	1.13	1.08	1.19	<0.0001
Verbal Memory	0.52	0.32	0.86	0.0109
Executive Speed	0.81	0.52	1.24	0.3215
Visual Memory	1.03	0.62	1.69	0.9216
Number Span	1.41	0.87	2.29	0.1578
Verbal Fluency	0.76	0.49	1.18	0.2249
BRIEF BRI				
Age (45–54 vs. 65–74)	0.98	0.26	3.76	0.9767
Age (55–64 vs. 65–74)	0.55	0.13	2.27	0.4078
Exposure (COL vs. PRO)	0.63	0.13	3.04	0.5633
Pre-existing attention disorder (Yes vs. No)	2.60	0.55	12.22	0.2260
Educational attainment	0.98	0.70	1.38	0.9010
Years of football play	0.96	0.82	1.13	0.6086
BDI Scale Score	1.19	1.12	1.26	<0.0001
Verbal Memory	0.54	0.31	0.94	0.0305
Executive Speed	0.51	0.32	0.83	0.0061
Visual Memory	1.36	0.78	2.36	0.2783
Number Span	0.59	0.34	1.00	0.0512
Verbal Fluency	0.76	0.47	1.22	0.2583
BDHI				
Age (45–54 vs. 65–74)	1.58	0.51	5.98	0.3747
Age (55–64 vs. 65–74)	1.62	0.65	5.87	0.4536
Exposure (COL vs. PRO)	2.14	0.60	7.96	0.2430
Pre-existing attention disorder (Yes vs. No)	0.57	0.12	2.63	0.4711
Educational attainment	0.98	0.27	1.35	0.9228
Years of football play	1.06	0.93	1.22	0.3689
BDI Scale Score	1.10	1.06	1.16	<0.0001
Verbal Memory	0.92	0.59	1.34	0.7124
Executive Speed	0.81	0.54	1.21	0.3089
Visual Memory	1.51	0.73	1.80	0.5494
Number Span	1.34	0.87	2.06	0.1790
Verbal Fluency	0.99	0.66	1.50	0.9738
CNS-LS				
Age (45–54 vs. 65–74)	0.37	0.12	1.12	0.0772
Age (55–64 vs. 65–74)	0.92	0.32	2.65	0.8784
Exposure (COL vs. PRO)	1.46	0.41	5.13	0.5572
Pre-existing attention disorder (Yes vs. No)	2.87	0.70	11.80	0.1432
Educational attainment	0.86	0.59	1.25	0.4354
Years of football play	1.10	0.97	1.25	0.1433
BDI Scale Score	1.08	1.03	1.12	0.0007
Verbal Memory	0.72	0.46	1.14	0.1576
Executive Speed	1.07	0.73	1.58	0.7322
Visual Memory	1.11	0.73	1.71	0.6219
Number Span	1.00	0.66	1.53	0.9860
Verbal Fluency	0.87	0.58	1.30	0.4999

*Cognitive function*. Among cognitive domains, better verbal memory performance was associated with reduced odds of impulsivity (BIS-11 OR = 0.52; 95% CI = 0.32, 0.86; *p*-value = 0.0109). Similarly, better verbal memory (BRI OR = 0.54; 95% CI = 0.31, 0.94; *p*-value = 0.0305) and executive speed (BRI OR = 0.51; 95% CI = 0.32, 0.83; *p*-value = 0.0061) were associated with reduced odds of behavioral dysregulation. None of the cognitive factor scores were independently associated with hostility/aggression (BDHI) or emotional lability (CNS-LS).

*Covariates*. Age group was independently associated with impulsivity, with participants aged 45–54 years demonstrating higher odds of elevated BIS-11 scores compared with those aged 65–74 (OR = 4.14; 95% CI = 1.06, 16.17; *p*-value = 0.0410). Age was not significantly associated with BRI, BDHI, or CNS-LS outcomes. Although the oldest age group (65–74) had significantly higher BDI scores than the youngest group (45–54) in the descriptive analyses (*p*-value = 0.0031), the interaction between BDI and age did not influence odds of elevated NBD scores. Exposure classification (PRO vs. COL), years of football participation, pre-existing attention disorders, and education were not independently associated with odds of elevated NBD scores across any outcome (Table 5).

### Exploratory factor analysis of depressive symptoms

Exploratory factor analysis of BDI items identified three distinct depressive symptom dimensions. These factors reflected (1) affective depressive symptoms, including sadness, discouragement, guilt, self-criticism; (2) neurovegetative features characterized by fatigue, sleep disturbance, appetite change, and reduced energy; and (3) agitated/reactive depression, including irritability, agitation, and crying.

When NBD scales were modeled as continuous measures, the three depressive factor scores demonstrated differential associations across domains. The neurovegetative factor score was the most consistently associated predictor across outcomes (Table 6). Higher neurovegetative scores were associated with greater impulsivity (BIS-11 estimate = 6.84; 95% CI = 4.45, 9.23; *p*-value < 0.0001), behavioral dysregulation (BRI estimate = 11.63; 95% CI = 8.50, 14.76; *p*-value < 0.0001), hostility/aggression (BDHI estimate = 5.07; 95% CI = 2.32, 7.81; *p*-value = 0.0004), and emotional lability (CNS-LS estimate = 1.16; 95% CI = 0.12, 2.20; *p*-value = 0.0284). The agitated/reactive factor score was independently associated with greater impulsivity (BIS-11 estimate = 3.42; 95% CI = 1.44, 5.41; *p*-value = 0.0008) and hostility/aggression (BDHI estimate = 5.11; 95% CI = 2.89, 7.32; *p*-value < 0.0001), but was not significantly associated with behavioral dysregulation or emotional lability. The affective factor score demonstrated more modest and outcome-specific associations. Greater affective depressive symptom burden was associated with increased behavioral dysregulation (BRI estimate = 3.09; 95% CI = 0.01, 6.18; *p*-value = 0.0494), but was not significantly associated with BIS-11, BDHI, or CNS-LS scales.

**Table 6:** Adjusted multivariate models incorporating depressive factor scores.

Effect	Estimate	95% CI		*p*-value
BIS-11				
Age (45–54 vs. 65–74)	4.41	0.24	8.58	0.0382
Age (55–64 vs. 65–74)	−2.20	−6.31	1.91	0.2918
Race (black/African American vs. white)	0.92	−3.90	5.74	0.7062
Exposure (PRO vs. COL)	−0.40	−1.44	0.65	0.4519
Educational attainment	−0.07	−0.56	0.42	0.7821
Years of football play	3.23	−2.53	8.99	0.2698
Pre-existing attention disorder (Yes vs. No)	−2.47	−5.98	1.05	0.1673
Verbal Memory	−2.02	−3.65	−0.40	0.0149
Executive Speed	−0.22	−1.77	1.33	0.7796
Visual Memory	−1.01	−2.62	0.59	0.2133
Number Span	0.61	−0.90	2.12	0.4272
Verbal Fluency	−1.42	−2.93	0.10	0.0662
Core depression factor score	1.97	−0.43	4.37	0.1071
Neurovegetative factor score	6.84	4.45	9.23	<0.0001
Agitative/reactive factor score	3.42	1.44	5.41	0.0008
BRIEF BRI				
Age (45–54 vs. 65–74)	−1.96	−7.37	3.45	0.4760
Age (55–64 vs. 65–74)	−3.94	−9.25	1.37	0.1449
Race (black/African American vs. white)	−0.97	−7.17	5.24	0.7583
Exposure (PRO vs. COL)	−0.31	−1.67	1.06	0.6557
Educational attainment	0.09	−0.55	0.72	0.7900
Years of football play	4.31	−3.22	11.83	0.2599
Pre-existing attention disorder (Yes vs. No)	−1.97	−6.55	2.61	0.3958
Verbal Memory	−2.64	−4.76	−0.52	0.0150
Executive Speed	−3.10	−5.11	−1.09	0.0027
Visual Memory	1.91	−0.16	3.97	0.0706
Number Span	−1.86	−3.83	0.11	0.0641
Verbal Fluency	−1.90	−3.88	0.08	0.0595
Core depression factor score	3.09	0.01	6.18	0.0494
Neurovegetative factor score	11.63	8.50	14.76	<0.0001
Agitative/reactive factor score	1.82	−0.77	4.42	0.1675
BDHI				
Age (45–54 vs. 65–74)	5.84	1.13	10.56	0.0155
Age (55–64 vs. 65–74)	1.51	−3.12	6.14	0.5195
Race (black/African American vs. white)	−2.58	−8.08	2.92	0.3556
Exposure (PRO vs. COL)	−0.94	−2.11	0.24	0.1167
Educational attainment	0.20	−0.35	0.76	0.4676
Years of football play	−2.62	−9.31	4.08	0.4413
Pre-existing attention disorder (Yes vs. No)	−1.26	−5.21	2.69	0.5289
Verbal Memory	−0.60	−2.42	1.22	0.5169
Executive Speed	−0.30	−2.05	1.44	0.7318
Visual Memory	1.53	−0.30	3.37	0.1013
Number Span	1.18	−0.52	2.88	0.1715
Verbal Fluency	0.89	−0.81	2.59	0.3009
Core depression factor score	1.76	−0.93	4.44	0.1976
Neurovegetative factor score	5.07	2.32	7.81	0.0004
Agitative/reactive factor score	5.11	2.89	7.32	<0.0001
CNS-LS				
Age (45–54 vs. 65–74)	−2.44	−4.25	−0.64	0.0084
Age (55–64 vs. 65–74)	−1.35	−3.13	0.44	0.1373
Race (black/African American vs. white)	−0.99	−3.08	1.10	0.3513
Exposure (PRO vs. COL)	−0.21	−0.66	0.24	0.3605
Educational attainment	0.16	−0.05	0.37	0.1370
Years of football play	1.05	−1.45	3.56	0.4059
Pre-existing attention disorder (Yes vs. No)	0.14	−1.38	1.67	0.8547
Verbal Memory	−0.66	−1.37	0.04	0.0648
Executive Speed	0.09	−0.59	0.76	0.7996
Visual Memory	−0.21	−0.90	0.49	0.5605
Number Span	0.26	−0.40	0.91	0.4425
Verbal Fluency	−0.13	−0.78	0.53	0.7007
Core depression factor score	0.76	−0.28	1.80	0.1497
Neurovegetative factor score	1.16	0.12	2.20	0.0284
Agitative/reactive factor score	0.52	−0.34	1.38	0.2319

Advancing age was associated with lower impulsivity and hostility/aggression, but higher emotional lability. Specifically, the youngest participants scored higher than the oldest participants on BIS-11 (estimate = 4.41; 95% CI = 0.24, 8.58; *p*-value = 0.0382) and BDHI (estimate = 5.84; 95% CI = 1.13, 10.56; *p*-value = 0.0155), whereas the oldest participants scored higher on CNS-LS (estimate = −2.44; 95% CI = -4.25, −0.64; *p*-value = 0.0084).

Domain-specific associations were found among cognitive factor scores and NBD outcomes. Better performance on verbal memory was associated with lower impulsivity (BIS-11 estimate = −2.02; 95% CI = −3.65, −0.40; *p*-value = 0.0149) and behavioral dysregulation (BRI estimate = −2.64; 95% CI = −4.76, −0.52; *p*-value = 0.0150). Better executive speed was selectively associated with lower behavioral dysregulation (BRI estimate = −3.10; 95% CI = −5.11, −1.09; *p*-value = 0.0027). No significant associations were observed among cognitive domains for hostility/aggression or emotional lability.

Exposure classification (PRO/COL), years of football participation, race, pre-existing attention disorders, and education were not independently associated with any NBD scale scores after adjusting for depressive factor scores (Table 6).

Among sensitivity analyses, no significant interaction between RHI exposure (years of football) and depressive symptom burden was observed for impulsivity, behavioral regulation, or hostility (See Table A2 in the Appendix). For emotional lability (CNS-LS), a significant interaction between RHI and depressive burden was identified (estimate = 0.02; 95% CI = 0.00, 0.03; *p* = 0.0301).

## Discussion

This study evaluated cross-sectional relationships among NBD, neurocognitive performance, depressive symptom burden, RHI exposure, and TES in former football players. Findings partially supported the study hypotheses. First, poorer neurocognitive performance was associated with greater NBD for select outcomes, although these relationships were not consistent across all scales. Greater RHI exposure was not independently associated with NBD after adjustment for depressive symptoms and other clinical factors. Second, depressive symptom burden was independently and consistently associated with greater NBD across all outcomes, supporting our hypothesis that depression contributes to neurobehavioral symptom burden. Third, depressive symptom burden modified the cross-sectional association between RHI exposure and emotional lability, but not other NBD outcomes. Together, these findings suggest that NBD is closely linked to depressive symptom burden, while cognitive performance and RHI exposure demonstrate more selective and context-dependent associations.

Depressive symptom burden emerged as the central factor underlying NBD, with consistent effects observed across impulsivity, behavioral regulation, hostility, and emotional lability. Neurovegetative depressive symptoms were the most strongly and consistently associated across all NBD outcomes, whereas agitated and affective depressive symptoms showed more selective associations. These findings extend prior work by demonstrating that the relationship between depression and NBD is not uniform and is associated with specific depressive syndrome dimensions (9). In contrast to depressive symptoms, cognitive performance demonstrated more limited and domain-specific associations with neurobehavioral outcomes. Better verbal memory was associated with lower odds of impulsivity and behavioral dysregulation, while better executive speed was selectively associated with lower behavioral dysregulation. No significant associations were observed between cognitive performance and hostility or emotional lability. These findings suggest that NBD is not a reaction to, or function of, global cognitive decline. These findings generate additional hypotheses that NBD and cognitive impairment may represent partially independent processes, warranting further research to identify the biological mechanisms underlying each.

Age was differentially associated with specific NBD outcomes. Younger participants demonstrated greater impulsivity and hostility, whereas older participants demonstrated greater emotional lability. This pattern suggests that NBD may not present as a uniform clinical phenotype across age. Rather, externalizing symptoms such as impulsivity and hostility may be more prominent among younger participants, while emotional lability may be more apparent among older participants and may overlap with behavioral features observed in other neurodegenerative conditions. One interpretation is that externalizing NBD symptoms may represent an early behavioral expression of affective dysregulation, whereas later-life symptom burden may shift toward less explosive but more emotionally labile presentations. Depression was strongly associated with NBD outcomes and was more prevalent among older participants, supporting the need to evaluate neurobehavioral symptoms and mood as related but clinically distinct contributors to symptom burden. Although COL participants demonstrated greater symptom burden than PRO participants across several NBD measures, younger age is unlikely to fully explain these differences. Age was associated with only select NBD domains, and playing level was not independently associated with NBD after adjustment for age, depressive symptoms, and other clinical factors. The observed PRO/COL differences therefore likely reflect multiple interrelated clinical and demographic factors rather than age or exposure classification alone. These findings should be interpreted cautiously given the cross-sectional design and the relationship between age and exposure history in this cohort, particularly because older participants were more likely to be former professional players with greater cumulative exposure.

In contrast, educational attainment was not independently associated with neurobehavioral outcomes after adjustment for depressive symptoms, implying that these relationships are not explained purely by differences in cognitive reserve. Exposure-related variables, including playing level and years of football participation, were also not independently associated with NBD across domains. Although descriptive differences were observed between exposure classification and TES status, these effects did not persist in adjusted models, suggesting that cumulative exposure alone does not account for variability in symptom burden. TES+ COL participants also demonstrated greater depressive symptom burden than TES+ PRO participants; however, the absence of an exposure × TES interaction suggests that, while PRO and COL differed overall, TES affected both groups in a similar way. Thus, the higher symptom burden observed among COL participants does not appear to represent a distinct TES-associated pattern specific to this exposure group. These findings challenge the assumption that NBD is primarily driven by exposure severity or reflects a direct consequence of cumulative RHI burden, as exposure-related variables were not independently associated with NBD after adjustment for age and other clinical factors. The observed findings suggest substantial inter-individual variability in behavioral outcomes following RHI exposure (9, 11, 30, 31).

Overall, the findings support a heterogeneous and multidetermined model in which NBD reflects the combined influence of mood, cognitive, and individual-level factors such as age. These findings further support the hypothesis that NBD is not simply a behavioral manifestation of cumulative injury or global cognitive decline, but a complex, phenotypic, multifactorial causation. The consistent and influential relationship between mood symptoms, especially neurovegetative changes, and NBD indicate the importance of assessing mood in individuals with RHI and manifesting NBD. Additional studies will determine if depression should be regarded as a core symptom of TES or should continue to be regarded as a supportive feature.

Several limitations should be considered when interpreting these findings. The sample consisted exclusively of former male American football players, which may limit generalizability to female athletes and other contact-sport or military-exposed populations. Although all neurocognitive tests were normalized to age and education, applying standardized reference values derived from the general population may have introduced misclassification, particularly given the lower average educational level of the PRO compared to the COL participants. This study is cross-sectional, and the data cannot quantify progression of the condition. Furthermore, the cross-sectional nature of these analyses precludes causal inference, and the reliance on self-reported exposure history may underestimate cumulative head impact burden. All participants in this study had substantial RHI exposure, limiting associations of clinical features with RHI to the upper range of exposures. Medication use and other treatments for depression or behavioral dyscontrol were not accounted for and may have influenced symptom severity in some participants. As recruitment into the study was based on football experience history and not on clinical features, individuals with behavioral or mood symptoms could have preferentially decided to participate.

Future studies should integrate longitudinal follow-up. Advanced multimodal modeling is necessary to clarify temporal trajectories of NBD and its relationship to evolving mood, cognitive, and biological markers. Expanding these methods to diverse forms of RHI exposure beyond American football will be essential to determine whether NBD represents a stable or pre-existing behavioral phenotype, a reversible functional state, or an early indicator of progressive neurodegeneration. A better understanding of these relationships may also help identify potentially modifiable symptom domains and therapeutic targets. With further characterization, this could inform future symptomatic clinical trials, including evaluation of existing or repurposed therapies.

## Conclusion

Among former American football players with substantial RHI exposure, depressive symptom burden was more consistently associated with NBD than football exposure characteristics or cognitive performance. Neurovegetative depressive symptoms demonstrated the broadest associations across impulsivity, behavioral dysregulation, hostility, and emotional lability, while cognitive performance showed more selective, domain-specific relationships. These findings support NBD as a heterogeneous clinical phenotype and emphasize the importance of evaluating depressive symptoms when interpreting NBD in the context of TES. Longitudinal studies incorporating biological, cognitive, and psychosocial measures are needed to clarify the temporal relationships among RHI exposure, depressive symptoms, NBD, and neurodegenerative disease.

## Data Availability

The datasets presented in this article are not readily available because they contain potentially sensitive participant-level data. De-identified data may be made available upon reasonable request and subject to applicable institutional, ethical, and data-use requirements. Requests to access the datasets should be directed to the corresponding author.

## Appendix

**Table A1:** NBD instrument clinical thresholds compared to empirical cut points.

Scale	Underlying mechanism	Reporting format	Clinical threshold	Empirical cut point
BIS-11	Trait impulsivity (attentional, motor, non-planning)	Raw score	65	58
BRI	Executive regulation (inhibit, shift, emotional control)	T-score	65	66
BDHI^*^	Hostility and aggression tendencies (overt and covert)	Raw score	-	28
CNS-LS	Pathologic affective lability (involuntary laughing/crying)	Raw score	13	15

^*^No clinical thresholds have been identified.

**Table A2:** Sensitivity analyses: interaction between RHI exposure and depressive symptoms across neurobehavioral outcomes.

Outcome	RHI × BDI interaction estimate*	95% CI		*p*-value
BIS-11	−0.03	−0.07	0.01	0.0903
BRI	0.02	−0.03	0.07	0.4028
BDHI	−0.02	−0.06	0.02	0.3248
CNS-LS	0.02	0.00	0.03	0.0301

^*^Estimates derived from multivariate regression models including RHI exposure (years of football participation), depressive symptom severity (BDI total), their interaction term, and covariates (age group, exposure classification, education, and cognitive factor scores). Interaction terms represent the product of centered variables.
